# Plastomes of the green algae *Hydrodictyon reticulatum* and *Pediastrum duplex* (Sphaeropleales, Chlorophyceae)

**DOI:** 10.7717/peerj.3325

**Published:** 2017-05-17

**Authors:** Hilary A. McManus, Daniel J. Sanchez, Kenneth G. Karol

**Affiliations:** 1 Department of Biological and Environmental Sciences, Le Moyne College, Syracuse, NY, United States of America; 2 Lewis B. and Dorothy Cullman Program for Molecular Systematics Studies, The New York Botanical Garden, Bronx, NY, United States of America

**Keywords:** Chlorophyceae, Plastome evolution, Green algae, Chloroplast genome, Hydrodictyaceae, Open reading frames, *Pediastrum*, Sphaeropleales, *Hydrodictyon*

## Abstract

**Background:**

Comparative studies of chloroplast genomes (plastomes) across the Chlorophyceae are revealing dynamic patterns of size variation, gene content, and genome rearrangements. Phylogenomic analyses are improving resolution of relationships, and uncovering novel lineages as new plastomes continue to be characterized. To gain further insight into the evolution of the chlorophyte plastome and increase the number of representative plastomes for the Sphaeropleales, this study presents two fully sequenced plastomes from the green algal family Hydrodictyaceae (Sphaeropleales, Chlorophyceae), one from *Hydrodictyon reticulatum* and the other from *Pediastrum duplex*.

**Methods:**

Genomic DNA from *Hydrodictyon reticulatum* and *Pediastrum duplex* was subjected to Illumina paired-end sequencing and the complete plastomes were assembled for each. Plastome size and gene content were characterized and compared with other plastomes from the Sphaeropleales. Homology searches using BLASTX were used to characterize introns and open reading frames (orfs) ≥ 300 bp. A phylogenetic analysis of gene order across the Sphaeropleales was performed.

**Results:**

The plastome of *Hydrodictyon reticulatum* is 225,641 bp and *Pediastrum duplex* is 232,554 bp. The plastome structure and gene order of *H. reticulatum* and *P. duplex* are more similar to each other than to other members of the Sphaeropleales. Numerous unique open reading frames are found in both plastomes and the plastome of *P. duplex* contains putative viral protein genes, not found in other Sphaeropleales plastomes. Gene order analyses support the monophyly of the Hydrodictyaceae and their sister relationship to the Neochloridaceae.

**Discussion:**

The complete plastomes of *Hydrodictyon reticulatum* and *Pediastrum duplex*, representing the largest of the Sphaeropleales sequenced thus far, once again highlight the variability in size, architecture, gene order and content across the Chlorophyceae. Novel intron insertion sites and unique orfs indicate recent, independent invasions into each plastome, a hypothesis testable with an expanded plastome investigation within the Hydrodictyaceae.

## Introduction

Organellar genomic studies of the green algae are revealing extensive variability in genome size, architecture and gene order, and phylogenomic analyses are resolving relationships and discovering novel lineages (Fučíková et al., 2014; Lemieux et al., 2015; Turmel, Otis & Lemieux, 2015; Fučíková, Lewis & Lewis, 2016a; Fučíková, Lewis & Lewis, 2016b; Leliaert et al., 2016; Lemieux, Otis & Turmel, 2016; Turmel et al., 2016). Of the five orders comprising the Chlorophyceae, the Sphaeropleales have garnered recent attention, with genomic studies characterizing dynamic evolutionary patterns in both chloroplast and mitochondrial genome architecture and gene content (Farwagi, Fučíková & McManus, 2015; Lemieux et al., 2015; Fučíková, Lewis & Lewis, 2016a; Fučíková, Lewis & Lewis, 2016b). Of these studies, only one has focused on genome evolution at the family level, analyzing the mitochondrial genomes of the Hydrodictyaceae (Farwagi, Fučíková & McManus, 2015).

The freshwater green algal family Hydrodictyaceae, a member of the Sphaeropleales and sister to the Neochloridaceae (Fučíková et al., 2014), includes the well-known genera *Hydrodictyon* Roth 1797 and *Pediastrum* Meyen 1829. The Hydrodictyaceae has undergone taxonomic revisions based on molecular phylogenetic studies of individual nuclear and chloroplast genes (Buchheim et al., 2005; McManus & Lewis, 2011); however, several relationships remain unresolved, particularly the paraphyly of *Pediastrum duplex* Meyen 1829 and its relationship to *Hydrodictyon* (McManus & Lewis, 2011). Farwagi, Fučíková & McManus (2015) presented the first complete mitochondrial genomes of four representatives from the Hydrodictyaceae. The results revealed size differences and gene rearrangements that carry phylogenetic signal, indicating that whole genome-level studies of the Hydrodictyaceae may be useful in resolving ongoing systematic questions.

To gain further insight into the evolution of the chlorophyte plastome and increase the number of representative plastomes for the Sphaeropleales, we fully sequenced the plastomes of a strain of *Hydrodictyon reticulatum* (L.) Bory 1824 and *Pediastrum duplex*. The complete plastomes of these Hydrodictyaceae strains, representing the largest of the Sphaeropleales sequenced thus far, once again highlight the variability in size, architecture, gene order and content across this order.

## Materials and Methods

*Hydrodictyon reticulatum* was collected from the freshwater Geyser Brook, Saratoga Co., NY, USA (43.058117, −73.807914) on 23 July 2014 and DNA was extracted directly from the field collection. A strain of *Pediastrum duplex* (EL0201CT/HAM0001) was isolated from the freshwater Eagleville Pond, Tolland Co., CT, USA (41.7848239, -72.2805262) in June 2002 and maintained in culture at 20 °C under a 16:8 h light:dark (L:D) cycle on agar slants. The agar slants consisted of a 50:50 mixture of Bold’s basal medium (BBM) (Bold, 1949; Bischoff & Bold, 1963) and soil water prepared following McManus & Lewis (2011) in 3% agar. Voucher material for each strain is deposited in The New York Botanical Garden William and Lynda Steere Herbarium (NY) under barcodes 02334980 and 02334981, respectively. Duplicate specimens of each are deposited in the George Safford Torrey Herbarium at the University of Connecticut (CONN) and in the personal collection of HAM.

Total genomic DNA was extracted from living cells following a CTAB extraction protocol (Doyle & Doyle, 1987). DNA was sent to the Woodbury Genome Center at Cold Spring Harbor Laboratories for TruSeq library preparation followed by sequencing on Illumina HiSeq2500 to produce 2 × 101 bp paired-end reads. Geneious v.9.1.5 (Biomatters, http://www.geneious.com) was used to trim, pair, and *de novo* assemble the reads. Several contigs containing plastome fragments were recovered for each strain after the initial *de novo* assembly. Geneious was then used to map reads to the plastome fragments in a series of reference assemblies until longer fragments were obtained that could be joined into a single sequence. DOGMA (Wyman, Jansen & Boore, 2004, dogma.ccbb.utexas.edu/), BLAST (http://blast.ncbi.nlm.nih.gov/), tRNAscan-SE 2.0 (Lowe & Chan, 2016), RNAweasel (Lang, Laforest & Burger, 2007, http://megasun.bch.umontreal.ca/cgi-bin/RNAweasel/RNAweaselInterface.pl), and Geneious were used to annotate each plastome. OrganellarGenomeDRAW (Lohse et al., 2013, http://ogdraw.mpimp-golm.mpg.de/) was used to draw plastome maps, and synteny maps were generated using the Mauve plugin (Darling et al., 2004) with default settings in Geneious. Gene order analyses were performed using MLGO: Maximum Likelihood Gene Order Analysis web server (http://www.geneorder.org/server.php) (Lin, Hu & Moret, 2013). BLASTX homology searches were used to characterize introns and open reading frames (orfs) ≥300 bp with an *E*-value threshold <1e − 06 (https://blast.ncbi.nlm.nih.gov/blast.cgi).

## Results

**Figure 1:**
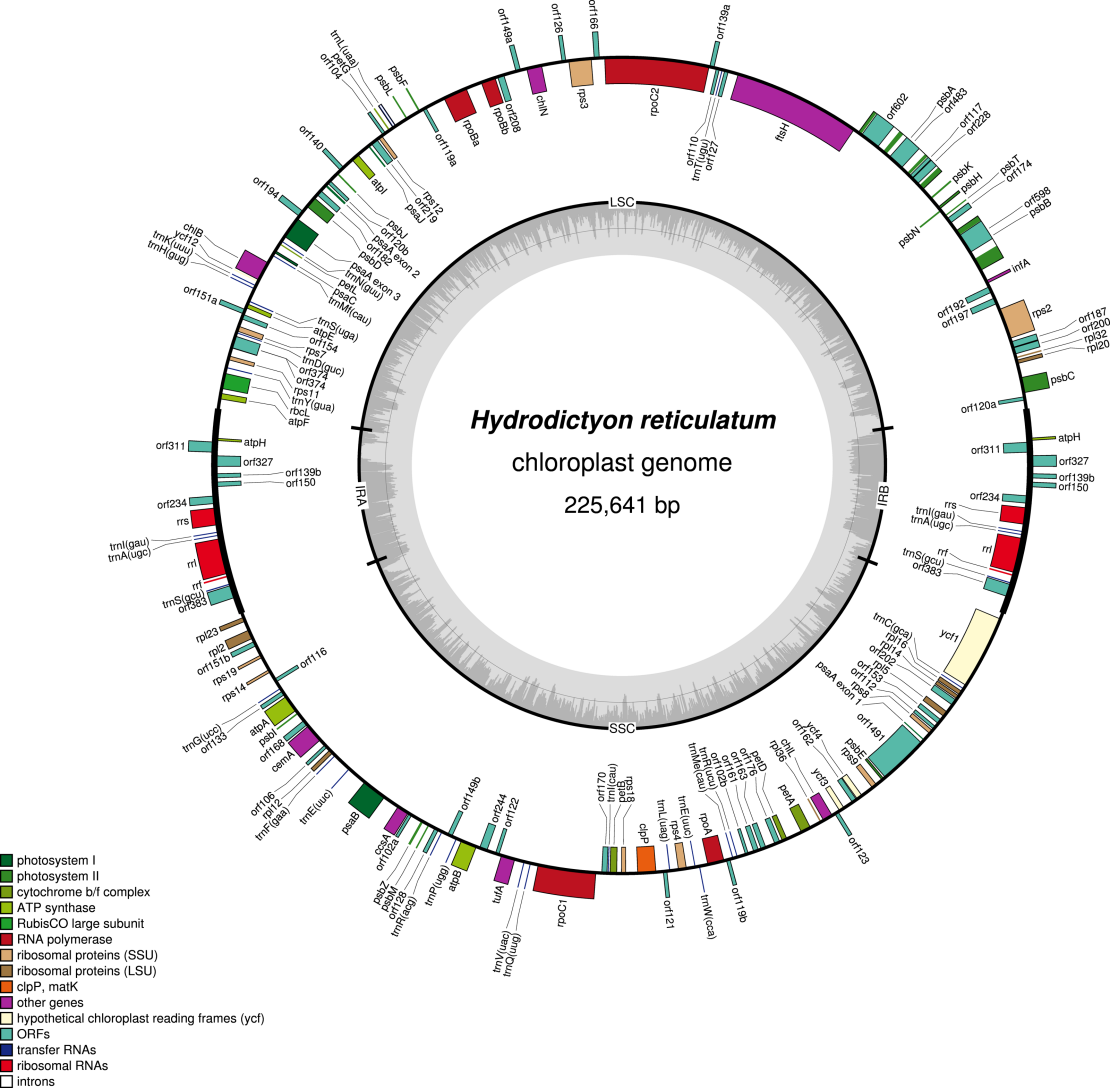
Gene map of *Hydrodictyon reticulatum* plastome (KY114065). The inverted repeats (IRA and IRB) which separate the genome into two single copy regions are indicated on the inner circle along with the nucleotide content (G/C dark grey, A/T light grey). Genes shown on the outside of the outer circle are transcribed clockwise and those on the inside counter clockwise. Gene boxes are color coded by functional group as shown in the key.

**Figure 2:**
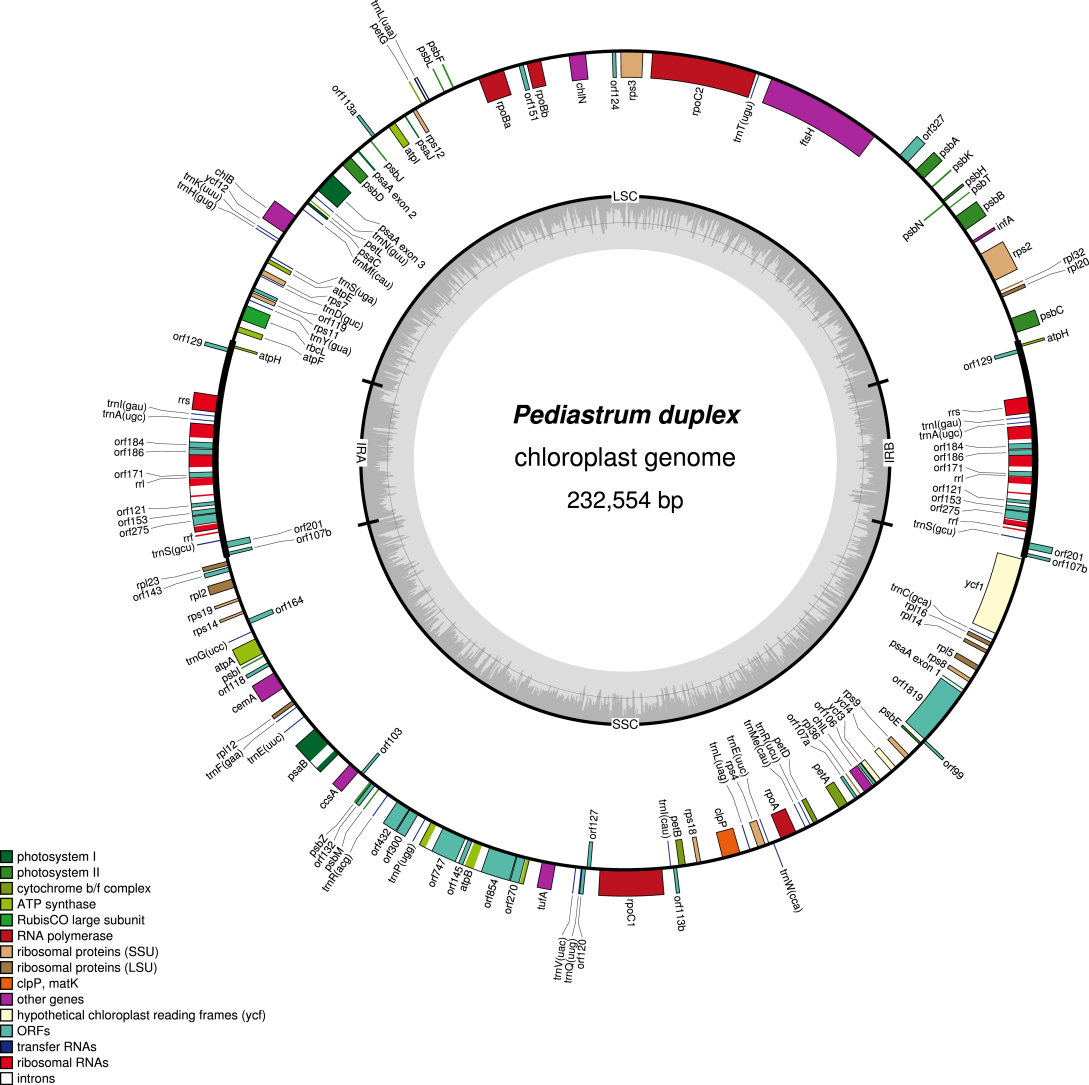
Gene map of *Pediastrum duplex* plastome (KY114064). The inverted repeats (IRA and IRB) which separate the genome into two single copy regions are indicated on the inner circle along with the nucleotide content (G/C dark grey, A/T light grey). Genes shown on the outside of the outer circle are transcribed clockwise and those on the inside counter clockwise. Gene boxes are color coded by functional group as shown in the key.

DNA sequence data collection resulted in 12.5 million paired-end reads for *Hydrodictyon reticulatum* and 9.9 million paired-end reads for *Pediastrum duplex*. The plastome for each strain was assembled with no gaps, and the average coverage was 195X (225,641 bp) for *H. reticulatum* (Fig. 1; GenBank accession KY114065) and 134X (232,554 bp) for *P. duplex* (Fig. 2; GenBank accession KY114064). Each plastome comprised two copies of an inverted repeat (IR) separated by two single-copy (SC) regions. *Hydrodictyon reticulatum* contained 102,823 bp and 86,226 bp SC regions and *P. duplex* 98,587 bp and 94,307 bp SC regions. Inferred protein translations indicated the universal genetic code was used in both plastomes, and RNA editing did not appear to be necessary. All protein-coding regions used the AUG start codon, with the exception of *psbC* that used GUG. The coding regions for each plastome included genes for 3 rRNAs, 25 unique tRNAs and 68 functionally identifiable protein genes, including *ycf1*, *ycf3*, *ycf4* and *ycf12* (Table 1). Fifty-nine putative open reading frames (orfs) ≥300 bp of unknown function were identified in the plastome of *H. reticulatum* and 32 were identified in the plastome of *P. duplex* (Table 1).

**Table 1:** List of plastid-encoded genes annotated for *Hydrodictyon reticulatum* and *Pediastrum duplex*. Open reading frames (orfs) ≥300 bp are indicated separately for each plastome.

Gene class	Genes			
Ribosomal RNAs	*rrf* x2IR	*rrl* x2IR * in Pd	*rrs* x2IR	
Transfer RNAs	*trnA-UGC* x2IR	*trnC-GCA*	*trnD-GUC*	*trnE-UUC* x2
	*trnF-GAA*	*trnG-UCC*	*trnH-GUG*	*trnI-CAU*
	*trnI-GAU* x2IR	*trnK-UUU*	*trnL-UAA* *	*trnL-UAG*
	*trnMe-CAU*	*trnMf-CAU*	*trnN-GUU*	*trnP-UGG*
	*trnQ-UUG*	*trnR-ACG*	*trnR-UCU*	*trnS-GCU* x2IR
	*trnS-UGA*	*trnT-UGU*	*trnV-UAC*	*trnW-CCA*
	*trnY-GUA*			
ATP synthase	*atpA*	*atpB* * in Pd	*atpE*	*atpF*
	*atpH* x2	*atpI*		
Chlorophyll biosynthesis	*chlB*	*chlL*	*chlN*	
Cytochrome	*petA*	*petB*	*petD*	*petG*
	*petL*			
Photosystem I	*psaA* ts	*psaB* * in Pd	*psaC*	*psaJ*
Photosystem II	*psbA* * in Hr	*psbB* * in Hr	*psbC*	*psbD*
	*psbE*	*psbF*	*psbH*	*psbI*
	*psbJ*	*psbK*	*psbL*	*psbM*
	*psbN*	*psbT*	*psbZ*	
Ribosomal proteins	*rpl2*	*rpl5*	*rpl14*	*rpl16*
	*rpl20*	*rpl23*	*rpl32*	*rpl36*
	*rps2*	*rps3*	*rps4*	*rps7*
	*rps8*	*rps9*	*rps11*	*rps12*
	*rps14*	*rps18*	*rps19*	
RNA polymerase	*rpoA*	*rpoBa*	*rpoBb*	*rpoC1*
	*rpoC2*			
Hypothetical proteins	*ftsH*	*ycf1*	*ycf3*	*ycf4*
	*ycf12*			
Miscellaneous proteins	*ccsA*	*cemA*	*clpP*	*infA*
	*rbcL*	*tufA*		
orfs (*H. reticulatum*)	*orf102a*	*orf102b*	*orf104*	*orf106*
	*orf106*	*orf110*	*orf112*	*orf116*
	*orf117*	*orf119a*	*orf119b*	*orf120a*
	*orf120b*	*orf121*	*orf122*	*orf123*
	*orf126*	*orf127*	*orf128*	*orf133*
	*orf139a*	*orf139b* x2IR	*orf140*	*orf149a*
	*orf149b*	*orf150* x2IR	*orf151a*	*orf151b*
	*orf153*	*orf154*	*orf161*	*orf162*
	*orf163*	*orf166*	*orf168*	*orf170*
	*orf174*	*orf176*	*orf182*	*orf187*
	*orf192*	*orf194*	*orf197*	*orf200*
	*orf202*	*orf208*	*orf219*	*orf228*
	*orf200*	*orf234* x2IR	*orf244*	*orf311* x2IR
	*orf327* x2IR	*orf374*	*orf383* x2IR	*orf483*
	*orf598*	*orf602*	*orf1491*^✜^	
orfs (*P. duplex*)	*orf99*	*orf103*	*orf106*	*orf107a*
	*orf107b* x2IR	*orf113a*	*orf113b*	*orf118*
	*orf119*	*orf120*	*orf121* x2IR	*orf124*
	*orf127*	*orf129* x2IR	*orf132*	*orf143*
	*orf145*	*orf151*	*orf153* x2IR	*orf164*
	*orf171* x2IR	*orf184* x2IR	*orf186* x2IR	*orf201* x2IR
	*orf270*	*orf275* x2IR	*orf300*	*orf327*
	*orf432*	*orf747*	*orf854*	*orf1819*^✜^

**Notes.**

ts trans-spliced * intron-containing gene in both plastomes * in Hr intron(s) in *H. reticulatum* but not *P. duplex* * in Pd intron(s) in *P. duplex* but not *H. reticulatum* X2 duplicated gene not in inverted repeat (IR) x2IR duplicated gene in IR ✜ shares 58.6% similarity between *H. reticulatum* and *P. duplex* (see Table 4)

The coding region made up 59.6% of the *Hydrodictyon reticulatum* plastome and 53.3% of the *Pediastrum duplex* plastome (Table 2). Gene content of known genes was similar to that of other Sphaeroplealean plastomes, but the *trnG* (gcc) gene was not detected in either plastome, similar to *Neochloris aquatica* (Fučíková, Lewis & Lewis, 2016a). The IR in *H. reticulatum* was 18,296 bp and contained *atpH*, *rrf*, *rrl*, *rrs*, *trnA* (ugc), *trnI* (gau), and *trnS* (gcu). The IR in *P. duplex* was 19,830 bp and included the same genes as *H. reticulatum,* plus an additional four introns in *rrl* not found in *H. reticulatum*. Like other members of the Sphaeropleales, *psaA* was trans-spliced in both plastomes with exon 1 in the smaller SC and exons 2 and 3 in the larger SC.

**Table 2:** Summary of *Hydrodictyon reticulatum*, *Pediastrum duplex* and other Sphaeropleales plastomes. %Coding includes all CDS (including orfs), tRNAs and rRNAs (both IRs); %GC content includes both IRs; Genes includes CDS (including orfs), tRNAs and rRNAs (both IRs).

Species	Strain	GenBank	Size (bp)	%GC	%Coding	Non-Coding (bp)	Genes	Introns	IR (bp)
*Acutodesmus obliquus*	UTEX 393	DQ396875	161,452	26.9	56.0	55,454	106	10	12,022
*Ankyra judai*	SAG 17.84	KT199255	157,224	28.3	57.0	64,708	109	2	8,247
*Bracteacoccus aerius*	UTEX 1250	KT199254	165,732	31.7	54.9	74,226	103	2	7,271
*Bracteacoccus minor*	UTEX B66	KT199253	192,761	31.9	48.4	96,439	104	3	9,577
*Chlorotetraedron incus*	SAG 43.81	KT199252	193,197	27.1	46.7	94,081	106	10	13,490
*Chromochloris zofingiensis*	UTEX 56	KT199251	188,937	30.9	47.2	97,693	106	2	6,375
***Hydrodictyon reticulatum***	**HAM0289**	**KY114065**	**225,641**	**32.1**	**59.6**	**91,268**	**111**	**5**	**18,296**
*Kirchneriella aperta*	SAG 2004	KT199250	207,516	34.1	42.3	76,270	106	27	35,503
*Mychonastes homosphaera*	CAUP H 6502	KT199249	102,718	39.8	80.1	20,264	105	1	6,472
*Neochloris aquatica*	UTEX 138	KT199248	166,767	30.3	50.9	54,026	102	32	18,217
***Pediastrum duplex***	**EL0201CT**	**KY114064**	**232,554**	**32.6**	**53.3**	**108,632**	**125**	**8**	**19,830**
*Pseudomuriella schumacherensis*	SAG 2137	KT199256	220,357	31.2	41.6	117,502	109	8	22,004

Two introns were present in *atpB* of *Pediastrum duplex.* Intron 1 contained two open reading frames (orf), one with a putative reverse transcriptase, intron maturase and HNH endonuclease (*orf145*) and the other with a reverse transcriptase with Group II origin (*orf747*) (Table 3). Intron 2 contained a reverse transcriptase of Group II intron origin (*orf854*). No introns were found in *atpB* of *Hydrodictyon reticulatum* (Table 3). *Pediastrum duplex* contained one intron in *psaB* that contained a putative GIY-YIG homing endonuclease, and both plastomes harbored an intron that lacked an orf in *trnL* (uaa). The *psbB* gene in *H. reticulatum* contained an intron housing a Group II intron reverse transcriptase (*orf598*). Three introns were present in *psbA* of *H. reticulatum.* The first contained a putative HNH homing endonuclease (*orf228*). The second and third intron each harbored a reverse transcriptase with Group II intron origin (*orf483* and *orf602*, respectively) (Table 3). Four introns were identified in *rrl* of *P. duplex* and not found in *H. reticulatum*. Intron 1 contained two putative site-specific DNA endonucleases (*orf184*, *orf186*), introns 2 and 4 each contained a LAGLIDADG superfamily homing endonuclease (*orf171* and *orf275*, respectively); intron 3 did not contain a detectable orf (Table 3).

**Table 3:** List of introns and contained conserved domains determined with BLASTX searches (*E*-value threshold < 1e − 06).

	*Hydrodictyon reticulatum*	*Pediastrum duplex*
*atpB* intron 1	–	*orf145*: putative reverse transcriptase, intron maturase and HNH endonuclease *orf747*: Reverse transcriptases (RTs) with group II intron origin (cd01651)
*atpB* intron 2	–	*orf854*: Reverse transcriptases (RTs) with group II intron origin (cd01651); Type II intron maturase (pfam01348)
*psaB*	–	no orf; putative GIY-YIG homing endonuclease
*psbB*	*orf598*: Reverse transcriptases (RTs) with group II intron origin (cd01651)	–
*psbA* intron 1	*orf228*: putative HNH homing endonuclease	–
*psbA* intron 2	*orf483*: Reverse transcriptases (RTs) with group II intron origin (cd01651)	–
*psbA* intron 3	*orf602*: Reverse transcriptases (RTs) with group II intron origin (cd01651)	–
*trnL* (uaa)	no orf	no orf
*rrl* intron 1	–	*orf184, orf186*: putative site-specific DNA endonuclease
*rrl* intron 2	–	*orf171*: LAGLIDADG DNA endonuclease (pfam00961)
*rrl* intron 3	–	no orf
*rrl* intron 4	–	*orf275*: LAGLIDADG DNA endonuclease family (pfam03161)

**Figure 3:**
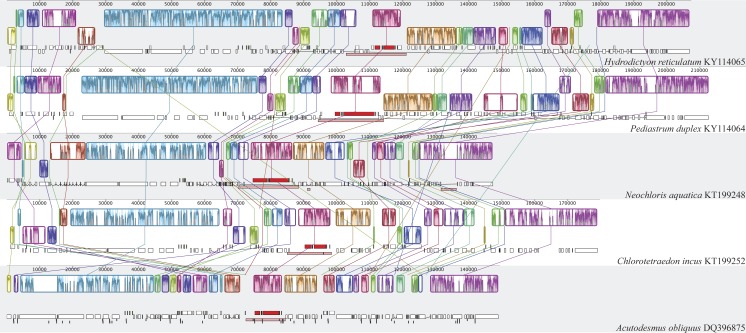
Synteny map of Hydrodictyaceae with Neochloridaceae and *Acutodesmus obliquus*. Blocks represent regions that align to a corresponding region in another genome and colored bars within each block indicate level of sequence similarity. Lines connecting blocks indicate putative homology.

**Table 4:** List of freestanding open reading frames ≥300 bp that harbor a conserved domain determined by BLASTX searches (*E*-value threshold < 1e − 06).

Taxon	orf	Conserved domain	Position
*Hydrodictyon reticulatum*	*orf374*	TolA protein (TIGR02794)	97334-96210
*Hydrodictyon reticulatum*	*orf244*	Putative reverse transcriptase, intron maturase and HNH endonuclease	152114-151380
*Hydrodictyon reticulatum*	*orf1491* (similarity with *orf1819* in *Pediastrum*)	Group II intron maturase-specific domain (pfam08388) putative reverse transcriptase and intron maturase (cl02808)	193836-189361
*Pediastrum duplex*	*orf300*	Replication-associated protein (McMurdo Ice Shelf pond-associated circular DNA virus-8) Sequence ID: YP_009047144.1	142962-143864
*Pediastrum duplex*	*orf432*	Replication-associated protein (McMurdo Ice Shelf pond-associated circular DNA virus-8) Sequence ID: YP_009047144.1	141638-142936
*Pediastrum duplex*	*orf1819* (similarity with *orf1491* in *Hydrodictyon*)	Group II intron, maturase-specific domain (pfam08388) Rft protein (pfam04506)	199224-193765

Multiple orfs greater than 300 bp were identified outside of intron regions, some of which contained HNH homing endonucleases or intron maturase proteins similar to those found in other green algae (Table 4). A reciprocal 50% protein similarity comparison of all orfs showed that none were shared between *H. reticulatum* and *P. duplex*, with one exception. The largest in both plastomes (*orf1491* in *H. reticulatum* and *orf1819* in *P. duplex*) shared 58.6% pairwise identity. Additionally, a 50% protein similarity search for each set of orfs with other complete sphaeroplealean plastomes resulted in no matches. In *Pediastrum duplex*, *orf300* and *orf432* each showed similarity with a virus replication-associated protein isolated from a freshwater pond on McMurdo ice shelf in Antarctica (circular DNA virus-8, YP_009047144, sequence similarity 1 × 10^−22^; Zawar-Reza et al., 2014).

**Figure 4:**
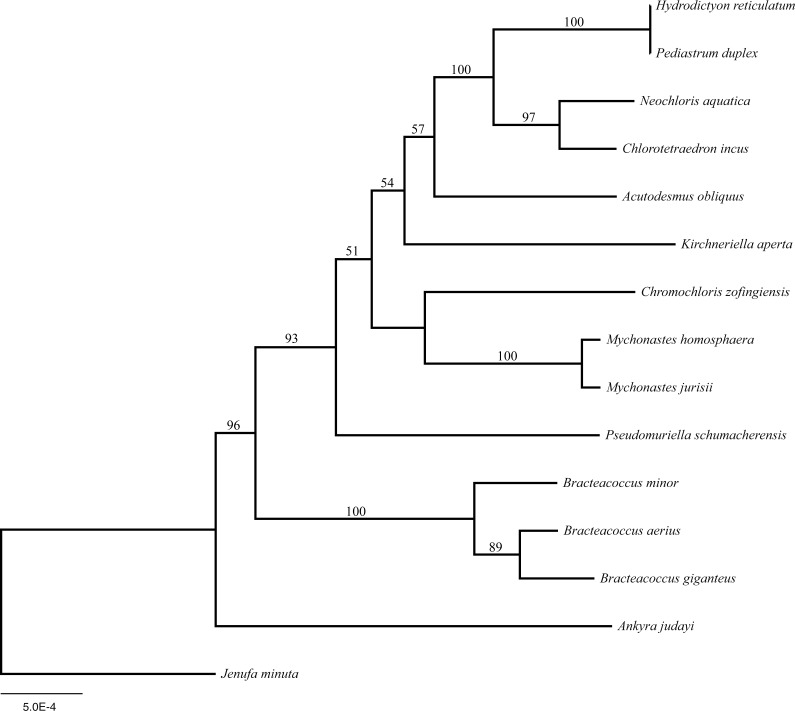
Maximum likelihood tree using plastome gene order within Sphaeropleales. ML bootstrap support values >50, based on 1,000 replicates, are indicated above each node.

Both plastomes shared identical gene order, while there were extensive rearrangements when compared with the closely related *Acutodesmus obliquus*, *Chlorotetraedron incus* and *Neochloris aquatica* (Fig. 3). The phylogenetic analysis of gene order recovered *Hydrodictyon reticulatum* and *Pediastrum duplex* as sister lineages with bootstrap support of 100, these in turn were found sister to a clade including *C. incus* plus *N. aquatica,* also with bootstrap support of 100. *Acutodesmus obliquus* was recovered sister to the above-mentioned taxa with bootstrap support of 57 (Fig. 4).

## Discussion

The addition of the two new Hydrodictyaceae plastomes permits a more rigorous analysis of plastomes across the Sphaeropleales, and highlights the importance of increased taxon sampling to aid in understanding plastome evolutionary trends. The plastomes of *Hydrodictyon reticulatum* and *Pediastrum duplex* were considerably larger in size compared with sister sphaeroplealean lineages *Neochloris aquatica*, *Chlorotetraedron incus* and *Acutodesmus obliquus*, and represent the largest plastomes thus far reported from the Sphaeropleales (Table 2). The size differences can be attributed to several factors, including relatively large intergenic regions (Table 2) and the infiltration of each plastome by numerous novel orfs (Table 3).

The relatively larger IR in the Hydrodictyaceae is consistent with the dynamic evolution of IRs discussed in Fučíková, Lewis & Lewis (2016b), and are larger than the ∼14 kb IR regions found in most fully-sequenced Sphaeropleales plastomes (with the exception of *Kirchneriella aperta* and *Pseudomuriella schumacherensis*), while similar to the IR found in *Neochloris aquatica* (∼18 kb). The IRs in *Hydrodictyon reticulatum* and *Pediastrum duplex* differ by 1,534 bp, and this difference is mainly due to the presence of four *rrl* introns in *P. duplex.* Presence and number of *rrl* introns across the Sphaeropleales does not appear to follow a clear phylogenetic pattern (see Fig. 1 of Fučíková, Lewis & Lewis, 2016a). This holds true for Hydrodictyaceae as well, but dense sampling within the family may uncover local phylogenetic patterns.

Intron number and distribution vary across the Sphaeropleales as well as within the Hydrodictyaceae. Of the five introns identified in *Hydrodictyon reticulatum* and eight introns in *Pediastrum duplex*, only the *trnL* (uaa) intron is shared by both. Six of the remaining 11 introns, one each in *atpB* and *psaB*, and two each in *psbA* and *rrl*, share identical insertion sites with other members of the order, suggesting possible ancestral origin of these introns. The last five introns have unique insertion sites in either *H. reticulatum* with two in *psbA* and one in *psbB*, or *P. duplex* with one in *atpB* and two in *rrl*. These introns with unique insertion sites could represent recent independent invasions into each plastome, a hypothesis testable with an expanded plastome investigation within the Hydrodictyaceae. The presence of a *trnL* (uaa) intron at base position 34 in both *H. reticulatum* and *P. duplex* is similar to other Sphaeroplealeaen plastomes, with the exception of *Ankyra judayi*, *Mychonastes homosphaera*, *Mychonastes jurisii,* and *Ourococcus multisporus* (Fučíková, Lewis & Lewis, 2016a). Based on available data, the phylogenetic distribution of this intron indicates that it is of ancestral origin and independently lost at least three times across the Sphaeropleales.

Many of the orfs 300 bp in size or larger and not located within an intron were identified as putative homing endonucleases and reverse transcriptases similar to those found in Group I and Group II introns (Table 4). The presence of these freestanding intron-like domains may indicate the translocation of *in situ* genic introns, or the invasion of intergenic spacer regions by novel elements (Turmel, Otis & Lemieux, 2015). Only two of the orfs (*orf1819* in *Hydrodictyon reticulatum* and *orf1491* in *Pediastrum duplex*) were similar to each other and not found in other sphaeroplealean plastomes, suggesting a common origin in the Hydrodictyaceae. The conserved maturase domain found in both suggests a functional importance in each plastome. The remaining orfs, 58 in *H. reticulatum* and 31 in *P. duplex*, were unique to each plastome. Because of their sister relationship in our study, we would expect to find homologous orfs if they were present in the common ancestor. Given the lack of shared orfs, it seems more likely that each lineage was independently invaded and that Hydrodictyaceae may be particularly susceptible to plastid viral infiltration. There is evidence that suggests chloroplasts are common targets of viruses (Li et al., 2016) and viral proteins have been reported in green algal plastomes of the Oedogoniales (Brouard et al., 2008), Trebouxiophyceae (Turmel, Otis & Lemieux, 2015), prasinophytes (Lemieux, Otis & Turmel, 2014; Turmel et al., 2009), and Zygnematophyceae (Lemieux, Otis & Turmel, 2016). *orf300* and *orf432* in *P. duplex* are the first report of genes putatively coding viral proteins in a plastome of the Sphaeropleales. Further analyses of sphaeroplealean plastomes are necessary to determine additional occurrences, functionality and origin of these novel orfs.

Four mitochondrial genomes of Hydrodictyaceae showed structural variability similar to that seen across the order (Farwagi, Fučíková & McManus, 2015). Thus far the structure of the plastomes is conserved between *Hydrodictyon reticulatum* and *Pediastrum duplex*, though additional plastomes within the family are anticipated to shed light on intrafamilial plastome evolution. The gene-order phylogenetic analysis presented here resulted in several well-supported relationships (Fig. 4) also recovered in individual gene and phylogenomic studies (Fučíková, Lewis & Lewis, 2016a; Fučíková, Lewis & Lewis, 2016b), indicating evolutionary relationships can be recovered using genome structure for this group. Incorporating additional Hydrodictyaceae (i.e., *Pseudopediastrum* and *Stauridium*) will determine if phylogenetic signal is reflected in plastome structure within the family.

## Conclusions

The plastome data reported here for two representatives from the Hydrodictyaceae, *Hydrodictyon reticulatum* and *Pediastrum duplex*, provide further insights into the evolution of plastomes in the Sphaeropleales and highlight plastome variability across the order. These plastomes represent the largest thus far sequenced from the Sphaeropleales, with the increased size being attributable to not only expansion of the IR and non-coding regions but also to infiltration of numerous novel open reading frames, many identified as putative homing endonucleases and reverse transcriptases, in both plastomes. Though both plastomes have acquired many orfs, the lack of similarity between these suggests independent acquisition in each lineage and further suggests a potential susceptibility of the hydrodictyaceaen plastome to invasion by novel elements. Phylogenetic analysis using plastome gene order in the Sphaeropleales is consistent with currently accepted phylogenetic schemes and provides an additional source of data for tree reconstruction across the order. More plastomes will need to be sequenced for the Hydrodictyaceae in order to test whether orf infiltration is common across the family or restricted to the *Hydrodictyon*/*Pediastrum* assemblage.

##  Supplemental Information

10.7717/peerj.3325/supp-1 Supplemental Information 1 Sequence data for *Hydrodictyon reticulatum* plastome (KY114065)

10.7717/peerj.3325/supp-2 Supplemental Information 2 Sequence data for *Pediastrum duplex* plastome (KY114064)

10.7717/peerj.3325/supp-3 Supplemental Information 3 Sphaeropleales plastome gene order dataset used for Fig. 4

## Additional Information and Declarations

### DNA Deposition

The following information was supplied regarding the deposition of DNA sequences: The plastome sequences described here are available via GenBank accession numbers KY114064 and KY114065. Sequence files are provided as Supplemental Data.

### Data Availability

The following information was supplied regarding data availability: The raw data have been supplied as Supplementary Files.
